# The Link between Mastery and Depression among Black Adolescents; Ethnic and Gender Differences

**DOI:** 10.3390/bs7020032

**Published:** 2017-05-12

**Authors:** Shervin Assari, Cleopatra Howard Caldwell

**Affiliations:** 1 Department of Psychiatry, School of Medicine, University of Michigan, 4250 Plymouth Rd., Ann Arbor, MI 48109-2700, USA; 2 Center for Research on Ethnicity, Culture and Health, School of Public Health, University of Michigan, 1415 Washington Heights, Ann Arbor, MI 48109-2029, USA; cleoc@umich.edu; 3 Department of Health Behavior and Health Education, School of Public Health, University of Michigan, 1415 Washington Heights, Ann Arbor, MI 48109-2029, USA

**Keywords:** population groups, ethnic groups, African Americans, youth, adolescents, depression, mastery

## Abstract

Purpose: Although the link between depression and lower levels of mastery is well established, limited information exists on ethnic and gender differences in the association between the two. The current study investigated ethnic, gender, and ethnic by gender differences in the link between major depressive disorder (MDD) and low mastery in the United States. Methods: We used data from the National Survey of American Life-Adolescent supplement (NSAL-A), 2003–2004. In total, 1170 Black adolescents entered the study. This number was composed of 810 African-American and 360 Caribbean Black youth (age 13 to 17). Demographic factors, socioeconomic status (family income), mastery (sense of control over life), and MDD (Composite International Diagnostic Interview, CIDI) were measured. Logistic regressions were used to test the association between mastery and MDD in the pooled sample, as well as based on ethnicity and gender. Results: In the pooled sample, a higher sense of mastery was associated with a lower risk of MDD. This association, however, was significant for African Americans but not Caribbean Blacks. Similarly, among African American males and females, higher mastery was associated with lower risk of MDD. Such association could not be found for Caribbean Black males or females. Conclusion: Findings indicate ethnic rather than gender differences in the association between depression and mastery among Black youth. Further research is needed to understand how cultural values and life experiences may alter the link between depression and mastery among ethnically diverse Black youth.

## 1. Introduction

According to the *differential effect/susceptibility theory* [1,2,3], race, ethnicity, gender, and place alter the mechanisms behind health and illness. In line with other theories that suggest that susceptibility to environmental influences depend on the context [4,5,6,7], the *differential effect theory* posits that complex interrelations between social, psychosocial, physical and mental health are not universal, but rather specific to sub- populations [8]. For instance, health effects of socioeconomic factors [1,2,9], mastery [10,11], and depression [12,13] all depend on race and ethnicity. Most of this literature is, however, derived from studies on adults, and less is known about similar heterogeneities in adolescents.

A large body of evidence has suggested stronger effects of depression on physical health outcomes for Whites than Blacks [14,15,16,17,18,19,20]. Depression, for example, increases the risk of chronic medical conditions [14,15] and all cause [15,16] and cause-specific [17] mortality among Whites, but not Blacks. Depression and depressive symptoms have also shown a correlation with expected biological markers, such as inflammatory markers in Whites but not Blacks [18,19,20]. Some evidence suggests that race and ethnicity may alter the health correlates of psychological and cognitive elements, such as sense of mastery, self-esteem and self-efficacy [10,11]. These psychological constructs appear to have stronger effects on mortality for Whites than Blacks [10,11].

The association is, however, less clear for the effects of depression on cognitive elements such as mastery and self-esteem. In agreement with the *differential effect (susceptibility) theory* [1,2,3,8], a number of recent studies have found evidence suggesting differential effect of depression on mastery and self-esteem (dysfunctional attitudes about self) across race and ethnic groups [10,12,13,21]. Racial and ethnic group differences have also been found in the effects of depression on hopelessness (dysfunctional attitudes about future) [22]. These studies cumulatively suggest that cognitive elements of depression are differently salient for each ethnic group. If these findings are true, they would advocate for tailoring psychotherapy and cognitive therapy for major depressive disorder (MDD) based on race and ethnicity [22]. However, most of the existing knowledge comes from studies of adults, as very few studies have ever examined these relationships in adolescents.

A particular theory may fail to explain behavioral and psychosocial phenomena across all social groups [23]. Researchers such as Lincoln [23] and others [24,25] have argued that social and psychological factors do not have universal effects across all ethnic groups, as distinct psychosocial processes may be involved in each sociodemographic group [23]. Due to the absence of previous studies on race/ethnic differences in the mechanisms of illness, however, researchers have traditionally assumed and searched for similar mechanisms across groups [26,27,28,29,30]. We argue that distinct social and psychosocial processes shape the physical and mental health of diverse populations, and this is a rule rather than an exception.

New information on ethnic differences in mechanisms that shape health can also shed light on the Black–White health paradox [14,15,31,32,33,34,35], defined as lower frequency of depression despite higher exposure to stress and adversities in Black than White adults [36,37,38,39,40,41,42,43]. More research is needed to understand origin of Black–White health paradox in childhood and adolescence.

Promotion of health and wellbeing of populations requires an understanding about group differences and heterogeneities in unique social and psychosocial processes that affect health and illness of diverse populations [23]. This argument is central to the *differential effect theory* [8]. It is, however, still unclear whether differential vulnerabilities that exist in adults [13,21] can be also seen in adolescents or not.

The current study investigated ethnic, gender, and ethnic by gender differences in the link between MDD and low sense of mastery among Black youth in the United States.

## 2. Methods

The present study used a cross-sectional design. Data came from the National Survey of American Life-Adolescent supplement (NSAL-A), 2003–2004 [44,45]. As a supplement to the NSAL-Adult study, the NSAL-A is one of the largest and the most updated mental health surveys of Black youth ever conducted in the U.S. [46,47,48]. Funded by the National Institute of Mental Health (NIMH), the study was a part of the Collaborative Psychiatric Epidemiology Surveys (CPES) series, and was conducted by the University of Michigan (UM) Institute for Social Research (ISR). More information on methodology of NSAL is available elsewhere [46,47,48].

### 2.1. Ethics

The UM Institute Review Board approved the NSAL (including NSAL-A component) protocol (B03-00004038-R1). All youths’ legal guardians provided informed consent. All youth provided assent. Participants received financial compensation ($50). All procedures were in accordance with the ethical standards of the institutional and/or national research committee. In addition, procedures abided to the 1964 Helsinki declaration and its later amendments or comparable ethical standards.

### 2.2. Participants and Sampling

A total number of 1170 Black adolescents were included in the NSAL-A. Participants were either African American (*n* = 810) or Caribbean Black (*n* = 360) youth who were 13–17 years old and lived in households with at least one Black parent in the United States at the time of survey. More information on the NSAL sampling and methodology is available elsewhere [47,48]. The NSAL used a national household probability sample of Blacks. To recruit the adolescent sample, all NSAL African American and Caribbean Black households were screened for eligible adolescents living in the same household. Adolescents were then randomly selected from all the listed families. If more than one eligible adolescent lived in the household, two adolescents would be selected based on the gender of the first selected adolescent. This strategy resulted in non-independence of the NSAL-A sample. To correct for this, the NSAL-A data were weighted to adjust for non-independence within the households and non-response rates at the households and individual levels. At the last step of sampling, the weighted data were post-stratified to represent the national estimates based on gender and age among African American and Caribbean Black youth [44,45].

### 2.3. Interview

About 82% of all interviews were conducted face to face, in the participants’ homes. The remaining 18% were conducted via telephone. Computer-assisted personal interview (CAPI) was used to enhance the quality of the data collected from in-person interviews. CAPI is one of the preferred interviewing techniques when the questionnaire is long and complex. In this technique, interviewers use computers to collect data. Interviews lasted on average 100 min, and were all performed in English language. The overall response rate was 81% (80% for African Americans and 83% for Caribbean Blacks).

### 2.4. Measures

The study measured demographic factors including age, gender and ethnicity. Socioeconomic status (family income) was measured as well. Clinical diagnosis of MDD was also measured using World Mental Health *Composite International Diagnostic Interview* (CIDI) [49,50].

#### 2.4.1. Ethnicity

Ethnicity was measured according to the parents’ self-identified ethnicity. Ethnicity of the parents who were living in the same household was assessed. Parents self-identified as African American if and only if they were Black and did not have ancestral ties to any Caribbean countries. Parents identified as Caribbean Black only and only if they were Black and they were from a Caribbean country. The list of Caribbean countries included: (1) Cuba, (2) Dominican Republic, (3) Haiti, (4) The Bahamas, (5) Jamaica, (6) Trinidad and Tobago, (7) Dominica, (8) Saint Lucia, (9) Antigua and Barbuda, (10) Barbados, (11) Saint Vincent and the Grenadines, (12) Grenada, and (13) Saint Kitts and Nevis.

#### 2.4.2. Major Depressive Disorder

Twelve-month MDD was measured using a modified version of the CIDI. A fully structured diagnostic interview schedule, the CIDI evaluates a wide range of Diagnostic and Statistical Manual, 4th Edition (DSM-IV) mental disorders including but not limited to MDD. Originally developed for the World Mental Health project initiated in 2000 [49], the CIDI is developed for use by trained lay interviewers to generate diagnoses of DSM-IV-TR/ International Classification of Diseases-10 (ICD-10) disorders [50]. Clinical reappraisal studies have documented acceptable psychometric properties such as good concordance of CIDI results with blinded clinical diagnoses [23,24,25,51,52]. This measure provides valid findings for Blacks and their ethnic groups [53,54,55,56,57,58]. In addition, this measure also has acceptable function in youth [59,60,61,62,63,64,65,66,67]. This outcome was treated as a dichotomous variable.

#### 2.4.3. Mastery

*Mastery* was measured using the Pearlin Mastery scale (adolescent version of the scale with 12 items). The scale measures the extent to which a person feels that they have control over events in his or her life [68]. Example items include: “There is really no way I can solve some of the problems I have”, “I often feel helpless in dealing with the problems of life”, and “What happens in my life is often beyond my control”. The item responses ranged from 1 to 4 as: (1) strongly disagree, (2) somewhat disagree, (3) somewhat agree, and (4) strongly agree. All items were reverse-coded. We calculated an average score. A higher score reflects higher feelings of control [69].

#### 2.4.4. Family Income

Family income was the socioeconomic factor in this study. Family income was mean-centered, and treated as a continuous measure.

### 2.5. Statistical Analysis

We used Stata 13.0 (Stata Corp., College Station, TX, USA) to account for the survey design of the NASL-A data. All the standard errors (SEs) were recalculated using the Taylor Series approximation technique to reflect design-based weights. All proportions reported in this study are weighted and representative of the U.S. population.

In the NSAL-A, the Caribbean Black sample is more clustered than the African American sample, SEs are systematically larger for Caribbean Blacks than African Americans. As a result, findings are more conservative in Caribbean Blacks than African Americans. This is in part because, while African Americans were sampled from large cities or other urban and rural areas, Caribbean Blacks were exclusively sampled from large cities.

Survey logistic regression models were used to conduct multivariable analysis. In our logistic regression models, 12-month MDD was the main outcome, sense of mastery was the predictor, and age, gender, ethnicity and SES were the control variables. Ethnicity and gender were also considered as effect modifiers. In the first step, the association of interest was estimated in the pooled sample, controlling for all confounders including gender and ethnicity. Then, in the next step, we estimated the same association of interest across ethnic, gender, and ethnicity by gender groups. Adjusted odds ratio (OR), SEs, 95% confidence interval (CI), and *p*-values were reported. A *p*-value of less than 0.05 was considered as statistically significant.

## 3. Results

### 3.1. Descriptive Statistics

Table 1 provides descriptive statistics for age, income, mastery and MDD in the pooled sample, and also based on the intersection of ethnicity and gender. Participants had an age of 15 years on average. The sample had an even distribution of males and females, however, most participants were African Americans.

### 3.2. Multivariable Model in the Pooled Sample

As Table 2 suggests, high sense of mastery was associated with lower risk of MDD in the pooled sample (Odds Ratio (OR) = 0.27, 95% Confidence Interval (CI) = 0.15–0.47). Age was also associated with risk of MDD (OR = 1.45, 95% CI = 1.17–1.79); however, ethnicity, gender, and family income did not have main effects on MDD risk.

Table 3 also shows a summary of two logistic regressions in males and females. High sense of mastery was associated with lower risk of MDD in males (OR = 0.16, 95% CI = 0.06–0.44) and females (OR = 0.42, 95% CI = 0.20–0.86).

### 3.3. Multivariable Models by Ethnicity and Gender

Table 3 shows a summary of two logistic regressions across ethnic groups. High sense of mastery was associated with lower risk of MDD in African Americans (OR = 0.23, 95% CI = 0.13–0.42) but not Caribbean Blacks (OR = 1.09, 95% CI = 0.16–7.62).

### 3.4. Multivariable Models in Ethnic by Gender Groups

Table 4 summarizes four logistic regressions based on the intersection of ethnicity and gender. High sense of mastery was associated with lower risk of MDD in African American males (OR = 0.15, 95% CI = 0.05–0.44) and African American females (OR = 0.36, 95% CI = 0.18–0.72) but not Caribbean Black males (OR = 1.16, 95% CI = 0.15–9.03) or females (OR = 1.03, 95% CI = 0.11–9.75).

## 4. Discussion

Using a national sample, we found a link between low sense of mastery and an increased risk of MDD among Black youth. Further exploration revealed that the link between sense of mastery and risk of MDD is only present in African Americans, but not Caribbean Blacks. Similarly, among African American males and females, but not Caribbean Black male or females, low mastery is linked to MDD risk.

Our finding regarding ethnic differences in the MDD-mastery link among Black youth aligns with previous studies showing that depression and depressive symptoms are differently associated with mastery and self-esteem in adults [13,21]. In a recent longitudinal study among older adults who participated in the Religion, Aging, and Health Survey, 2001–2004, high level of depressive symptoms at baseline predicted a greater decline in sense of mastery over a three-year follow-up period among Whites but not Blacks. Similarly, among Whites but not Blacks, low mastery at baseline predicted an increase in depressive symptoms over time [12]. In a cross-sectional study among older adults, a stronger association was found between low self-esteem and high depressive symptoms among Blacks compared to Whites [13]. In another paper using the NSAL-Adult data, the association between MDD and self-esteem was stronger in Black males than White males; however, the association did not differ in White and Black women [21]. Thus, ethnic groups differ in the link between evaluation of self (e.g., mastery and self-esteem). It is still not clear whether depression leaves a larger scar on self-evaluation for Blacks, or if Blacks with poor evaluation of self are more vulnerable to depression. We are not aware of any previous studies on the association between MDD and mastery in Black youth. Thus, there is a need for studies on racial, ethnic, and gender heterogeneities in the health effects of social and psychosocial resources [70,71,72,73,74,75,76,77]. The unique contribution of the current study is extending a well-established literature in adults to youth.

Our findings help us better understand ethnic differences in psychosocial and medical correlates of depression [14,15,16,17,51,70,71,72,73,74,75,76,77,78], as well as health consequences of low sense of control, self-efficacy, and mastery [10,11]. All these studies collectively suggest that the effects of risk and protective factors are specific to sociodemographic groups. This is in part because context and life conditions shape vulnerabilities and susceptibilities. The actual mechanism behind *differential effect/susceptibility theory* is, however, unclear [1,2,3,8].

As low mastery is poor evaluation of the self, these findings expand the existing knowledge regarding the implications of race and ethnicity for Beck’s theory. Based on this theory, depression is composed of dysfunctional evaluation of the self, others and future [52]. It has been previously shown that race alters how depression is associated with dysfunctional evaluation of the self, others [13,21], and future (hopelessness) [22]. Thus, race, ethnicity, and culture alter how depression distorts evaluation of the self, others and the future.

Our findings may have clinical and public health implications. Low sense of mastery may be a more salient cognitive element of depression for some groups than others. These findings can inform the design and evaluation of psychotherapy for MDD in diverse populations [22]. Specifically, cognitive treatment of MDD should address and accommodate differential cognitive profile of diverse depressed patients.

Such race and ethnic group variations may be due to group differences in historical experiences and cultural values that cumulatively shape resilience and vulnerability of populations to risk and protective factors. Thus, the very same exposure to a social or psychosocial factor would have differential health consequences across groups, as people of various groups differently respond and cope, and have different resources to buffer the stress.

The finding of this study adds support to race and ethnic differences in how psychosocial resources (e.g., appraisal, coping and mastery) correlate with depression. According to the *differential effects theory*, the health effects of psychological assets such as mastery, self-efficacy and self-esteem are specific to race and ethnicity [51,68,69,79,80,81,82,83,84,85,86,87,88]. This theory is supported by a number of studies showing that race and ethnicity modify social [81,82] as well as health correlates of psychological assets and depression [7,14,15,68,69,70,71,76,84,85].

Race and ethnic differences in vulnerability and resilience to risk and protective factors may be a consequence of chronic and lifelong exposure to psychosocial risk factors in ethnic minorities that begin early in life [17,51,89,90]. Another explanation is ethnic differences in the nature and meaning of depression or mastery [16]. Social and psychosocial construct do not reflect similar aspects of health across diverse groups [51,91].

As racial and ethnic differences are not limited to exposure but also vulnerabilities, at least some of the racial and ethnic disparities in health may be due to the indirect effects of race and ethnicity (not due to differential exposure but differential vulnerabilities). Ethnicity and race have contextual impact on health, in which they alter availability and efficacy of social and psychological resources and assets that can be used to buffer the effect of stressors when a single or a combination of risk factors occur [14,70,73,84,91,92,93].

Differential link between MDD and mastery across ethnic groups may explain why depression and mastery differently predict health of race and ethnic groups [17,88,89,90,91,94,95]. In a consistent manner, regardless of the type of the predictor and outcome, social and psychological factors have differential effects on physical and mental health outcomes for diverse populations [89,90,95,96]. Mastery and depression are not exceptions to this rule.

Our study is subject to a number of limitations. First, we used a cross-sectional design, so our findings suggest associations not causations. Second, our study is subject to measurement bias, as MDD and mastery may have differential validity across groups. Third, we had residual confounding, as we also did not measure history of anti-depressant use or health care use, which alter course of depression. Forth, sample size was not balanced across ethnic by gender groups. Fifth, the sample was more clustered for Caribbean Black youth. Despite all these limitations, our study was one of the firsts to explore ethnic and gender variation in the links between mastery and MDD in youth. Moreover, using a national sample was the other strength of this study.

To conclude, even among Black youth, ethnicity may modify the association between sense of mastery and risk of MDD. This finding supports the previous research on racial differences in the link between depression and mastery among adults and elderly. MDD may not similarly accompany dysfunctional attitudes about self, others and future across race and ethnic groups. It is still unknown why racial and ethnic differences exist in the paths between depression and self-evaluation (e.g., mastery). Future research should test whether cultural values, social class, life experiences, or socialization alter how mastery relates to depression across diverse sociodemographic groups.

## 5. Conclusions

In summary, findings of the current study indicate ethnic but not gender differences in the association between depression and mastery among Black youth. Additional research is needed to understand how culture and life experiences alter the bidirectional associations between depression and mastery among Black youth.

## Figures and Tables

**Table 1:** Descriptive statistics in the pooled sample and based on the intersection of race and gender.

	All		African American Males		African American Females		Caribbean Black Males		Caribbean Black Females	
	Mean (SE)	95% CI	Mean (SE)	95% CI	Mean (SE)	95% CI	Mean (SE)	95% CI	Mean (SE)	95% CI
Age	14.97 (0.061)	14.85–15.09	14.82 (0.098)	14.61–15.03	15.54 (0.054)	15.43–15.66	14.82 (0.098)	14.61–15.03	15.54 (0.054)	15.43–15.66
Family Income (Centered)	0.00 (2.12)	−4.29–4.29	−0.05 (3.00)	−6.20–6.09	−0.02 (2.45)	−5.04–5.00	1.77 (4.20)	−7.23–10.77	−0.51 (3.93)	−8.95–7.92
Mastery	3.16 (0.02)	3.12–3.20	3.19 (0.02)	3.14–3.23	3.14 (0.03)	3.08–3.20	3.05 (0.04)	2.97–3.14	3.21 (0.03)	3.14–3.28
MDD 12 Months										
No	95.74 (0.01)	94.33–96.81	95.56 (0.01)	93.30–97.09	96.06 (0.01)	93.63–97.58	97.74 (0.00)	96.79–98.41	92.41 (0.02)	86.76–95.76
Yes	4.26 (0.01)	3.19–5.67	4.44 (0.01)	2.91–6.70	3.94 (0.01)	2.42–6.37	2.26 (0.00)	1.59–3.21	7.59 (0.02)	4.24–13.24

MDD = Major Depressive Disorder; SE = standard error; CI = confidence interval.

**Table 2:** Summary of logistic regression in the pooled sample.

	OR (SE)	95% CI
Ethnicity (Caribbean Black)	1.07 (0.28)	0.63–1.80
Gender (Female)	0.94 (0.32)	0.48–1.87
Age	1.45 (0.15) ***	1.17–1.79
Family Income ($1000)	1.00 (0.00)	1.00–1.01
Mastery	0.27 (0.08) ***	0.15–0.47

Outcome: 12-Month Major Depressive Disorder. OR = odds ratio. *** *p* < 0.001.

**Table 3:** Summary of logistic regressions in ethnic groups.

	OR	95% CI	OR	95% CI	OR	95% CI	OR	95% CI
	African Americans		Caribbean Blacks		Males		Females	
Ethnicity (Caribbean Blacks)	-	-	-	-	0.46 (0.13) **	0.26–0.82	1.63 (0.60)	0.77–3.44
Gender (Female)	0.86 (0.32)	0.41–1.82	2.08 (1.08)	0.69–6.32	-	-	-	-
Age	1.42 (0.16) **	1.13–1.79	1.74 (0.48) #	0.96–3.16	1.50 (0.28) *	1.03–2.18	1.38 (0.17) *	1.07–1.78
Family Income ($1000)	1.00 (0.00)	1.00–1.01	0.99 (0.01)	0.97–1.01	1.01 (0.00) **	1.00–1.01	0.99 (0.01)	0.98–1.00
Mastery	0.23 (0.07) ***	0.13–0.42	1.09 (0.99)	0.16–7.62	0.16 (0.08) ***	0.06–0.44	0.42 (0.15) *	0.20–0.86

Outcome: 12-Month Major Depressive Disorder. # *p* < 0.1, * *p* < 0.05, ** *p* < 0.01, *** *p* < 0.001.

**Table 4:** Summary of logistic regressions in ethnic by gender groups.

	African American Males		African American Females		Caribbean Black Males		Caribbean Black Females	
	OR	95% CI	OR	95% CI	OR	95% CI	OR	95% CI
Age	1.47 (0.28) #	1.00–2.17	1.37 (0.19) *	1.03–1.81	2.20 (0.87) #	0.95–5.12	1.65 (0.59)	0.76–3.56
Family Income ($1000)	1.01 (0.00) **	1.00–1.01	0.99 (0.01)	0.98–1.01	1.01 (0.03)	0.95–1.08	0.98 (0.01)	0.96–1.01
Mastery	0.15 (0.08) ***	0.05–0.44	0.36 (0.12) ***	0.18–0.72	1.16 (1.11)	0.15–9.03	1.03 (1.08)	0.11–9.75

Outcome: 12-Month Major Depressive Disorder. # *p* <0.1, * *p* < 0.05, ** *p* < 0.01, *** *p* < 0.001.
